# Having, making and feeling home as a European immigrant in the United Kingdom post‐Brexit referendum: An interpretative phenomenological study

**DOI:** 10.1111/bjso.12798

**Published:** 2024-09-06

**Authors:** Kate Foxwell, Sarah Strohmaier, Fergal Jones, Dennis Nigbur

**Affiliations:** ^1^ School of Psychology and Life Sciences Canterbury Christ Church University Canterbury UK; ^2^ Present address: Gloucestershire Hospitals NHS Foundation Trust Gloucester UK; ^3^ Present address: Institute for Health and Sport, Victoria University Melbourne Victoria Australia

**Keywords:** acculturation, identity, interpretative phenomenological analysis, meaning of home, migration

## Abstract

Migrants' subjective sense of home deserves further research attention. In the particular context of the United Kingdom's (UK's) decision to leave the European Union (‘Brexit’), we interviewed 10 European citizens living in the UK about their sense of home, using interpretative phenomenological analysis (IPA). In our analysis, we identified themes of (1) having more than one home, (2) making and finding a new home, (3) being permanently different from the non‐migrant population and (4) a concern about feeling safe and welcome. Migration and sense of home involved building and rebuilding personal and social identity. Making a new home was effortful, and neither the old home nor the difference from the native population ever disappeared psychologically. This adds an experiential aspect to the idea of ‘integration’ in acculturation. Different notions of home were linked to different experiences of the impact of the Brexit referendum. We discuss the connections between acculturation, sense of home and lived experience and propose lived identity as a fruitful subject matter for social psychology.

## BACKGROUND

Whereas ‘house’ is an architectural concept, ‘home’ is a psychological one (Valsiner, 2014, p. 186): A sense of home is about whether a person *feels* at home in a particular space. This feeling is linked to place identity—the aspects of a person's identity that develop in relation to physical environments (Proshansky, 1978). Therefore, a person's sense of home and identity can change during and after international migration (see Romoli et al., 2022). The study reported here examines the phenomenology of home among European immigrants to the United Kingdom (UK), where the host country's departure from the European Union (EU) has added complexity.

Although a sense of home is necessarily personal and subjective, permeated by the feelings and meanings that people attach to the places they call home, it can also have a collective aspect, when a community to which the person feels attached is associated with a place. Place identity and social identity (Tajfel & Turner, 1979) may therefore be linked. Among social identities, national identity is especially territorial because nationalism seeks the congruence of people, place and political structure (Salazar, 1998). The country, as the ‘home’ in national identity, is often invoked to make arguments about identity and intergroup relations (Abell et al., 2006) and to claim ownership and jurisdiction (Verkuyten & Martinovic, 2017). Places, meanings and identity are thus intertwined at both individual and collective levels in the idea of a home or homeland, which deserves further understanding. This paper connects the psychological acculturation literature (Berry, 1997) with investigations of a sense of home (Nowicka, 2007) and recent phenomenological studies about how people live, and make sense of, their social identities as nationals and migrants (Warren & Nigbur, 2024).

### Home and international migration

A sense of home is of particular interest in international migrants, with the move to a different country bringing individual experiences of home into focus (Romoli et al., 2022). Mobility challenges the idea of home, and home is continuously constructed around a person's interactions and relationships (Nowicka, 2007). On the other hand, research on transnationalism may have neglected stability: Both ‘routes’ and ‘roots’ are involved in migrants' sense of home, with the meaning of home shifting and evolving, while simultaneously remaining remarkably unchanged (Ralph & Staeheli, 2011). Even Nowicka's (2007) transnational participants, who moved frequently for work and for whom moving was part of their lifestyle, tried to create a home as some kind of focal point, albeit temporary. The stability of home may thus be a defining characteristic, which is modified– not nullified – by migration.

Among international migrants, the sudden need to interact with unfamiliar authorities, a new culture and potentially a new language, may engender acculturative stress (see Berry et al., 1987) and raise questions about the nature of home. With both forced and voluntary migration, settling in a new country is associated with intensive reflection on identity (Andreouli & Howarth, 2013; Ballentyne et al., 2021; Timotijevic & Breakwell, 2000), and continued identification with one's ethnic heritage usually supports the wellbeing and mental health of migrants (Nesdale & Mak, 2003; Phinney et al., 2001). These findings show that migration and identity are intertwined, but do not explicitly cover the notion of home as something that could connect the two.

### Acculturation and intergroup relations

Berry's (1997) influential acculturation framework posits four different orientations, defined by whether the individual desires (a) to maintain their heritage culture and (b) to engage in meaningful contact with other cultural groups and participate in mainstream society. An orientation where both heritage culture maintenance and meaningful intergroup contact are pursued (‘integration’ in Berry's taxonomy) is hypothesised to be the most adaptive in most circumstances. There is substantial supporting evidence from migration studies (Berry et al., 2006; Choy et al., 2021), but also several criticisms about measurement (Rudmin, 2009), the invisibility of individual, first‐person experiences (Chirkov, 2009) and a neglect of the role of the receiving culture (Schwartz et al., 2010). Acculturation is an intergroup process (Berry, 1997; Bourhis et al., 1997; Brown & Zagefka, 2011), but little is known about how the perceived orientation of the host culture, and any changes in that stance, affect the lived experience of immigrants—despite some evidence from quantitative studies that intergroup perceptions of acculturation orientations can influence participants' own attitudes (Celeste et al., 2014; Matera et al., 2012).

Qualitative approaches have investigated migrant perspectives more specifically. Barker's (2015) participants talked about adopting cultural aspects of the countries between which they migrated, after noticing and evaluating cross‐cultural differences. Discourse analyses of immigrants' talk about integration have highlighted how people spoke about difficulties in the receiving country (Goodman et al., 2014), domains and possibilities of acculturation (Anjum et al., 2018), and hate crime (Kirkwood et al., 2013), among others. However, none of these studies has focused on participants' sense of home in their acculturation experiences.

### EU citizens in the UK post‐Brexit

European Union (EU) citizens living in the United Kingdom (UK) are particularly interesting in this respect. The UK leaving the EU after a referendum in 2016 (‘Brexit’) fundamentally changed the relationship between the UK and the EU, and the status of EU citizens living in the UK. EU citizens had previously been relatively invisible within the UK (Rzepnikowska, 2019) but became a controversial campaign focus preceding the referendum (Hobolt, 2016). Recent research (Botterill & Hancock, 2019; Hall et al., 2022; Racz, 2020; Ranta & Nancheva, 2019) has considered the experiences of some of the circa 4 million EU migrants living in the UK (Migration Observatory, 2024). Since the Brexit referendum, their sense of belonging—which is related to a sense of home (Antonsich, 2010)—has been shaken, due to potential changes not only to their rights but also to their identities (Guma & Jones, 2019). A large‐scale survey of EU citizens in the UK post‐Brexit (Bueltmann & Bulat, 2021) showed Brexit to constitute a rupture in participants' reasoning about their long‐term future in the UK. Feeling ‘in between’ belonging and not belonging was found to be a common experience among young European immigrants to the UK (Tyrrell et al., 2019). This is the context in which we examined how EU citizens make sense of *home* in the UK.

### Analysing lived experience

Interpretative phenomenological analysis (IPA; Smith et al., 2022) has become popular in health psychology to explore subjective lived experiences. Recently, it has also been used in social psychology to study migration, multiculturalism, and how people experience and make sense of their social identities in these contexts (‘lived identity’: Hunt et al., 2021; Warren & Nigbur, 2024). Interpretative phenomenological analysis is phenomenological in its focus on how people experience and make sense of phenomena from their own unique perspectives, and interpretative in going beyond a description of these perspectives towards a better understanding of the phenomenon. Interpretative phenomenological analysis uses a characteristic double hermeneutic, with researchers trying to make sense of a participant making sense of experience. This necessitates a detailed idiographic engagement with each participant's perspective before taking the analysis to the cross‐case level. Interpretative phenomenological analysis captures both convergence and divergence among the experiences of different people to understand the phenomenon. Instead of using large samples to draw potentially generalisable conclusions, IPA aims for highly detailed engagement with a small number of participants to yield valid insights that may be transferable to similar cases.

Interpretative phenomenological analysis is well suited to exploring how participants experienced and made sense of home, migration and life as an EU citizen in the UK post‐referendum. We aimed to contribute to research about the situation of EU citizens in the UK post‐Brexit (see above), extend academic understandings of the concept of home among migrants (Ralph & Staeheli, 2011), add first‐hand perspectives to the study of acculturation with sensitivity to local and personal contexts (Chirkov, 2009), and demonstrate the usefulness of IPA in studying how people live, experience and make sense of their social (Tajfel & Turner, 1979) and place identity (Proshansky, 1978).

## METHOD

### Design

We conducted qualitative research using IPA (Smith et al., 2022) to design the study and analyse data, and used semi‐structured interviews to collect data. The interviews included questions about home, country of origin, life in the UK and experiences of wider societal factors linked to participants' sense of home.

### Participants

Participants were recruited through social media, using opportunity and snowball sampling in 2017, approximately 1 year after the referendum, and before the UK officially withdrew from the EU in 2020. We interviewed 10 EU citizens aged 25 years and over, who had chosen to live in the UK and considered, at least at one point, that the move could be permanent. All had resided in the UK for at least 5 years, been employed for at least 1 year of that time and did not have British citizenship.

Participants' pseudonyms and basic demographic information are given in Table 1. All participants identified as white Europeans. They had lived in their countries of origin for 17–35 years. One participant had lived in the UK for 5 years, and the remaining nine for more than 10 years, two of them for more than 40 years. Six of the 10 had lived in other countries also. Three participants were single, two were in long‐term partnerships, four were married and one widowed. Occupations ranged from skilled manual work to those requiring postgraduate study. Two participants were retired or semi‐retired.

**TABLE 1:** Participant pseudonyms, countries of origin and basic demographic information.

Pseudonym	Origin	Gender	Age group
Hanna	Sweden	Woman	70s
Markus	Germany	Man	70s
Kristina	Czech Republic	Woman	50s
Alessia	Italy	Woman	30s
Chrysa	Greece	Woman	30s
David	France	Man	30s
Francesca	Italy	Woman	30s
Julija	Lithuania	Woman	30s
Lucie	Czech Republic	Woman	30s
Tomas	Slovakia	Man	30s

*Note*: More detailed demographic information has been omitted to preserve anonymity. Where relevant, participants' backgrounds are elaborated in the analysis.

### Data collection and ethics

The study was approved by a university ethics panel and adhered to British Psychological Society guidelines. All participants provided informed consent. Interviews took place in person in the South East of England and were audio‐recorded for subsequent transcription. Travel expenses were reimbursed and vouchers given as thanks for participation. Debriefing was carried out after each interview.

### Analysis and quality assurance

Smith et al.'s (2022) approach was followed. Individual case analyses, including personal experiential themes and their explanations alongside interview quotations to illustrate and justify them, were written for all 10 participants and independently audited. Cross‐case analysis then examined convergence and divergence between these idiographic case analyses to develop group experiential themes. Due to the wealth and complexity of data, these themes were further developed as aspects of superordinate group themes. The connections between themes were tracked diagrammatically. This part of the analysis, too, was audited to ensure that group themes were clearly based on all participants' data. The research team openly discussed each case study as well as the cross‐case analysis to ensure coherence and quality. Two members of the research team are EU citizens in the UK (although the interviewer is British), so we were especially careful to ensure that themes were transparently grounded in our participants' experiences rather than our own.

## RESULTS

Participants' sense‐making seemed to centre on ideas of having, making, and feeling at home as a migrant in the UK. A sense of home was partly serendipitous, but importantly also the product of conscious effort. Experiential themes at the cross‐case level illustrate overlapping but distinct aspects of this (see Table 2): Participants usually felt that they had more than one home, reasoned about both making and finding home, understood themselves as permanently different from non‐migrants and showed a concern for feeling safe and welcome in their new home.

**TABLE 2:** Overview of group experiential themes.

Superordinate theme	Subordinate theme (level 1)	Subordinate theme (level 2)
Having more than one home	Home is separate from nationality, culture and identity, and influential to them	Problematic or complex concept for migrants
		Different homes with different meanings
	Plant new seeds, keep old roots	Home as a place and a feeling
		Knowing home by comparison: cultural preferences persist
		Making new roots: choice and commitment
		Childhood home never goes away
Making and finding home	Feeling at home, feeling like a guest and sometimes both	Making new roots: choice and commitment
		Personal qualities as a homemaker
	Temporary moves become long‐term homes	Permission to enjoy and feel at home
		Communities and families make homes (old and new)
		Opportunities for growth
		Choosing versus happening to be somewhere
	Creating an adult home	
Being permanently different	Insider/outsider engagement with national cultures	
	Being a guest (rather than a native) is a starting point and default position	No promised land: international migration as a life choice
Concern with feeling safe and welcome	Making a home will fail if unwelcome	Losing a new (emotional) home after Brexit
		Feelings of connection with family, friends and good neighbours
	Belonging and identifying in diversity	London's diversity and community: somehow separate from England and the UK as a whole
		Brexit casts migrants as outsiders and seems incompatible with respected British values
		Brexit may or may not be a threat to sense of self and home

### Having more than one home

Having moved internationally, participants shared a sense that they had at least two homes. However, there were differences in how these homes featured in participants' thoughts, feelings and sense of identity. Nationality, culture and identity were not used as synonyms for home, but a sense of home was involved in all of them. Participants were aware that the concept of home was complex for them and attached different meanings to different homes. Home was not just a place to them, but also a feeling.

For example, Markus made a clear distinction between his physical, chosen home in the UK and his emotional homeland in Germany. Practical decisions around work and family had brought him to the UK and continued to keep him there, but affection and yearning persisted for the home of his formative years. To Markus, feeling familiar and happy with his situation in the UK was not the same as identifying as British:

I know home is here physically, with my wife is here, my children, my grandchildren are here, I'm happy here, my work was here, but emotionally I'm still German. I have not taken out UK citizenship, but so emotionally I'm still feeling German and although my brother and sister still live in Germany, since my parents died maybe eight/nine years ago, the link is becoming less strong because I've gone less often. But, if you were to ask me, where I'm at home, I, I can't say. It's sort of 50/50. I can't emotionally see that the land is … when I travel through Dorset let's say, or somewhere, or Norfolk, the land is not my land. It is not my homeland.

Intellectually and practically, Markus ‘knows’ that his home is in the UK and that he is happy where he lives. He also reasons about the link with Germany becoming weaker with the death of his parents and less frequent visits. But he repeats that he feels German, is emotionally German and can neither see his current residence as his homeland nor himself seeking citizenship. This distinction is apparent throughout his interview but is not experienced as a straightforward resolution: ‘I can't say. It's sort of 50/50’.

The complexity of a sense of home seems to be a common experience, but different participants made sense of their different homes with different meanings in different ways. Hanna's story is similar in some ways to that of Markus, as she moved to the UK to be with her husband, managed to fit in well, and stayed because of family. Sweden and being Swedish remain important to her through practical ties including family, friendships and property ownership, but also through a profound affection for the Swedish culture, landscape, and even the light:

Yeah I think it's the light in Sweden you know it's a sort of erm it's it's the open spaces but it's also light erm so erm I I do feel when I come back here you know that it's all very dark and and erm that's what I miss you know the sort of wherever you are in Sweden and I'm incredibly lucky because I've got you know all my friends live in wonderful houses with a lot of light and you know sea views and yeah so it's erm it's the it's and then to be able to go out in the forest and walk or go and walk around the coast yeah is absolutely wonderful.

Visiting Sweden made Hanna feel as if she had never left (‘I never really felt that I emigrated here you know, I never really felt that I left’). There are also times when she feels neither Swedish nor English, which she surmises must be common among people like her—‘most of the time it's okay but sometimes you feel slightly you know erm slightly like an outsider without being an outsider if you understand what that is?’ Having more than one home may thus mean feeling fully identified with none of them.

Francesca, meanwhile, reflected mostly on the distinction between her childhood and adult homes. Similar to Markus, she repeatedly mentioned feeling a ‘fifty‐fifty’ split between her Italian roots and the adult home she has made for herself in London. Both remain important aspects of her life: She feels settled in her job and social life, while simultaneously continuing to identify as Italian and appreciating Italian culture (reflecting the aforementioned distinctions between home, nationality, culture and identity). Francesca mentioned that her original home was currently perhaps losing emotional as well as practical significance, but still remains ‘back home’:

Having my job as well, because in Italy I don't think I could find anything that pays as well or you know, it's given me opportunities to travel, to have my mortgage. So yeah, I just feel like I'm really settled here. I have new friends here, my friends back home have kind of moved anyway from the village where I grew up, so although I see them when I go back, but I'm never back long enough to see everyone. My social life is here so again, it feels more … or if I go on holiday, I travel with friends I met here now, not so much the people I have back home. So to me it feels more home to be in London. Of course Italy is my roots and I can go there and relax, it's quiet, but I feel I don't belong there any more.

Notably, some participants had more complex migration histories and more than two places featuring in their narratives. But it remained the case that different homes held different meanings and that the original home, where people had spent their childhood, featured prominently in one way or another. David, who had spent the last decade living in four different countries and professed no intention to stay in the UK forever, talked throughout his interview about moving as a way of learning and evolving (‘when you travel you learn twice faster or you learn so many more things’), but was clear about the continued significance of his French background:

David London for me it's like my home now but there is something that will always stay, like, that I will always call France home as well. So I will … I don't know for what reason I am French, I was born in France and France will always be my home.

Interviewer Mmm.

David So maybe you can have different … many homes?

Recognizing the complexity and different meanings that the concept of home held for them, participants showed awareness of home as both a place and a feeling, and made comparisons between several homes and non‐homes to make sense of these feelings. The childhood home was portrayed as something that never goes away, whereas the current home was a place where participants more actively sought to establish themselves; and this sometimes resulted in a sense of home (see ‘Making and finding home’ below). Sometimes the idea of growth was invoked in describing this process, including metaphors about growing plants. Kristina half‐joked that ‘home is where the allotment is’, while Lucie made a similar reference about planting new seeds and preserving roots:

I think it's something to do with roots and planting seeds (laugh). So I think after moving to the UK, it took, it took me a while to consider this a home and it hasn't been my home for maybe six or seven years. But recently I have kind of, now I feel like I have two home, I have the Czech home and then this home too.

Lucie's words underline how the realization of more than one home is a process that takes time. Here, she talks about several years before she started considering the UK her home; elsewhere, she mentions how her husband helped her feel settled and how she eventually allowed herself to enjoy the new place of residence and make new roots: ‘Allowing myself to enjoy England was what allowed me to get more rooted here I think’. A sense of home in the new environment was thus characterized as being akin to making new roots. In Lucie's case, this involved letting go of a sense of anger at the new home, which she attributed to fear of losing her Czech identity—that is, her identification with the old home:

Lucie I had a lot of anger towards England when I moved here, which was very strange, because I wanted to move here. Little things would just drive me nuts like um … having two taps in the bath (laughs) and one of my legs be hot and one cold and just tiny things and like why are people doing it like this, you know, driving on the left and just tiny things like that that I would somehow project all my upset onto but I think that was just to do with leaving home was harder than I expected.

(…)

Interviewer Can you say anything more about the anger, how you made sense of that?

Lucie Erm I think I was just scared. I think I was scared that there were a lot of things that I enjoyed and that that would make me less Czech and very English.

Giving herself permission to enjoy was instrumental in accepting the new place as home, since the enjoyment had previously seemed like a threat to Lucie's being Czech. This idea of moving on was prominent throughout Lucie's interview: She moved on from the idea that she had to stay with her family physically because she was needed, from the idea to find one single place to call home and from worries about not feeling Czech enough.

Other participants talked about making a choice and commitment to a new, additional home. Kristina mentioned being anxious, even after many years of living in the UK and having a family, about telling her mother about her allotment—because it implied that she was settling in her new place of residence and not coming back. Markus and Lucie both described defending the UK in conversations with people from their original homes, which is testament to this commitment.

With the childhood home remaining a permanent feature of the participants' lives, it was common to feel a strong pull to return because of specific life crises or simply getting older. Kristina, for example, does not seem to believe that she will live in England permanently. Repeatedly, she feels that she is ‘packing my bags’ to return. There are various ‘push factors’—marital difficulties, unsatisfying work circumstances and not feeling wanted as a European citizen after Brexit are all mentioned—but the ‘pull factor’ is exerted by Kristina's old home in the Czech Republic:

Erm I really didn't want to leave, I had very comfortable life to sort of well‐organised from my point of view. And erm but because he [Kristina's husband] honestly asked me and erm you know, it was his daughter it felt just I have no rights so I thought I'm coming for a couple of years, max. He thought it would be a couple of months that it won't last. And then of course things get complicated (laughs) and my friends are laughing that I'm still, I still keep you know every year I'm packing my bags so talking about it that I'm going back, so what's my home? It's I I haven't resolved it in many ways.

Kristina describes her old life as very comfortable and the decision to move as one where she felt she had little agency. Going back to that old home is something she was expecting to do, and something she feels like doing when frustrated by the new home. She talks about not managing to ‘resolve’ those two places, which may imply that she belongs to both in some way, and to neither completely.

Having several homes, then, is partly a privilege and partly a source of doubt, complexity, and occasionally unease. Different homes fulfilled different functions. The psychological commitment to the new home seems to be crucial in accepting it as home, but this commitment is never absolute and sometimes questioned.

### Making and finding home

International migration, in the lives of our participants, was a deliberate choice rather than a forced displacement. This choice was not necessarily to find a new home (in the psychological sense), but the process of feeling at home occurred as the participants started to see both a present and a future in the new place.

For Alessia, this involved a memorable disruption of, and departure from, her old home, and an imagining of a new home that only happened considerably later. She described her old home and living arrangements, having to care for her grandmother with dementia, as a traumatic experience:

And then the last month, I could smell the smell of death. Describing how it is, I don't know, but I could smell it and I can still smell it. And so when my [grand]mother died, I ran away basically. But it's not because my parents were mean to me, it's just I was suffocating.

Alessia uses visceral language to describe the constraints of the old life (‘suffocating’) and its sudden end (‘the smell of death’). Her grandmother's death was a point of departure for her to start living her own life—an opportunity to escape this crushing responsibility. Still, family remained important to Alessia. Having left the old home, she started feeling at home in the new place when she began to see a family life on her own terms being created:

I think I started settling once I met my you know, current husband. Yeah about five years ago I met him and when I started realising that my family could actually be here, then it start feeling like this was my home.

A sense of home in the new environment, then, was something to which the participants made active contributions, creating a psychological sense of home after migration, as well as making a physical home. Some linked this to growing up and having their own ‘adult’ home. For Alessia, this was in having her own family, while for Francesca, her migration was linked to a sense of freedom and independence:

I never wanted to rely on anyone or a man to provide. I don't know, I don't have this ideal that from the village I suppose that you have to get a man, you have to be a housewife. I was like, really? Why? But I was kind of seen as the black sheep. I had friends, it wasn't a problem, but they were always like oh, not even the first few years when I used to go back more often, everyone kept saying, but don't you miss it here, are you coming back? And I was like, oh I don't think so. And the more I stayed in London, the more I found it hard to go back because I'd see the same faces in the same place.

The new home was a coming‐of‐age experience in that it was Francesca's own, and clearly distinguished from the past home and its expectations. Similarly, Julija spoke about becoming an adult and self‐reliant with the move to the new home. She acknowledged that leaving the parental home and forging her own path was associated with a sense of loss—a division of the original family and a loss of support. Ultimately, however, this was understood as a necessary part of becoming an independent adult:

Yeah, moving from parental home to that independence, so it was like nobody here around you, you're just really by yourself in here and nobody helps you. You know that you cannot just like (pauses) ring your mum and say oh Mum, I'm coming. It's just like you know, I need to deal with everything by myself. So you're growing up stronger and you starting like you know just to, you know you don't have nobody to rely on and to ask money or how to ask this. So you're growing up a personality and I think why I feel like a bit more grown up in here, I'm just like as an adult.

This highlights both the challenge and the rewards of international migration, the latter here characterized as personal growth. Some participants like Julija, Francesca or Lucie foregrounded the aspect of becoming an independent individual; others mentioned work opportunities that they took because they felt they had to (Tomas) or because they wanted to develop (David). Participants commonly talked about how having a family and becoming integrated in the local community was a milestone where the move became more long‐term, and a place to live became a home. Tomas was very clear about his home being where his young family is:

Yes, my new family now … It's, well I I'm still going to love my dad and mum, that's never going to change. But now my heart is getting more love for [baby] and [partner], because they're here. I I miss my parents, I try to get home every year, but sometimes it's not just happening like that, it's I don't have enough money for that. But yeah this is this is home, that's it. It's just, I can't think different now because I can say that, it's just … I I miss my parents, but they must realise that well I got a new family here, I got my own baby so that's the separation.

Here, Tomas connects the notion of home to the notion of love for those who share it. His love for his parents persists (and he still talks about going back ‘home’ every year), but the love that connects him to his partner and child creates a new home. The idea of a temporary move becoming permanent with family was a prominent theme in Tomas's interview. In the extract above, this seems to be blended with the coming‐of‐age motif also used by other participants. More than others, though, Tomas felt that his sense of home was defined *only* by his family:

No, this is there's nothing really important apart from my family, it's it's nothing important in the country for me. So I'm not going to miss anything from here if maybe‐ friends who I met through all the years and depends when we move. If I'm going to move someone else, it's a bit of a different people, I'm probably going to miss this like friendship with the people and erm calm people and talking about anything during the day if you meet someone, depends. If it's still here, I'm not giving like … erm no no, nothing attachment like oh, I have to stay here because of this. No.

In Tomas's view, then, families make homes. His birth family and his upbringing in Slovakia mean that Slovakia is home in his heart. His new family makes him feel at home in the UK, but he would also move elsewhere with them. Home is obviously where the family is, and the family being together is what matters in his home.

The local community was also mentioned as something that made participants feel at home. A sense of being supported and integrated was experienced as instrumental in building a sense of home. Julija's local community helped her settle after migration and again after the Brexit referendum:

12 years we're in the same place, so it's just like we have a community in here now. The way things are most days like you know everybody here. All the college, the ladies from the college have made me to go to the college to study as well. Let's go with us, we're going to go to the college.

Here, being invited to classes makes a positive difference to feeling settled and at home. Francesca also felt that community with friends in the new home gradually created a sense of home, while simultaneously the sense of home attached to the old place was diminishing with friends moving away from her native village (see above).

There were also cases where this active psychological home‐making did not happen. Hanna never sought a new psychological home. She never felt the need to commit to the new place because, to her, the move was always about following her husband and being with the family. Her choice was for the relationship, not for the place where they happened to live.

Hanna I never really felt that I emigrated here you know, I never really felt that I left as such, so maybe that is erm it.

Interviewer And why do you feel like you never left?

Hanna Erm I I think I started to think about that you know erm in relations to all the the people that are forced to leave their country erm I you know, it just happened, I you know I I never really intended to come here erm so maybe because of that I never really felt that I left as such you know, I'd‐ there wasn't this big question of immigrating, leaving my erm my home country really, I just came and I just stayed and it, I was happy to be here.

In the initial 6 months after Hanna went to London, she kept her flat in Sweden. Having such a grounding in Sweden was an important safety net, although she showed her intention to stay in London in other, more practical ways, such as bringing her own bed from Sweden—a symbol of intention to live this new life, even if her sense of home remained bound up with family rather than place. Hanna reasoned that her situation was relatively easy because she felt ‘comfortable’ in the new place, having been easily accepted by the community. She wonders whether ‘Maybe this whole idea of home is not is not really that important to me’. This seems to echo Tomas, who was very open to the idea of moving with his family because they sufficiently defined ‘home’ in his mind.

The gradual and often slow process of developing a sense of home had its limits for many participants and was not always entirely successful. It was common to feel at home, feel like a guest or sometimes both. For Chrysa, as for many participants, the move was prompted by following family—her sister in her case. She explained how comparisons with the life she had or might have had in her original home were often associated with a sense of disappointment in the new place:

I was comparing so much the life between here and my friends I had at home that I was constantly miserable, you know, it's like [sighs] I can't believe it's like, you know when you have these expectations of what something would be like and it wasn't.

Over time, she managed to establish a sense of home tied to particular homely places and people in London. This coexisted with a broader sense of home attached to her place of origin:

So whenever I go and see them, that's definitely about home, you know I can go there anytime, I don't have to tell them or something like that. So that's somewhere else I feel like a home in London. So that's I think that's sometimes the thing that's happened or or my best friend [name], when I go to his house, it feels like home. So I feel like in London I've created these little mini homes and then I have this kind of a broad home whenever I go back to Crete, so that's something else that yeah, I would consider that I guess.

This remarkable solution of ‘mini homes’ underlines that the sense of home is a personal and creative effort and requires homemaking skills as well as time. Francesca recognizes this challenge and points towards some personal qualities that may be required to rise to it:

So I'm very sociable but I'm also like really good by myself. Which is fine, I don't know, I think I'm just really strong and really tough, so London hasn't put me down even when I've had problems at work before I got [my job]. I just get on with things, I'm really practical, I'm really, nothing is a problem, it's a situation and we've got to, you know (laughs), deal with it. But I've realised that it's not for everyone. People have come to London or why would you do it? And kind of gone back, the people I know from my area.

Making and finding a home is explained as an active process, helped by other people such as families and welcoming communities, but ultimately relying on the commitment and skills of the individual. Starting with a move for practical reasons, people may gradually develop a sense of home and see their own future in the new place—or, sometimes, with particular people. But a sense of having more than one home, and being a guest in the new place, does not necessarily seem to disappear over the years.

### Being permanently different

Even if they had created a sense of home in the UK, participants felt different from the native population, permanently and inevitably. This was not necessarily seen as a bad thing: It gave the participants the privilege of an insider–outsider perspective on the adopted home and its culture, including the ability to choose how to blend the cultures of several homes. For some, like Julija, this was associated with the opportunity for a new life different from the one she had lived in her place of origin. She repeated in various forms throughout the interview that she found Lithuania today to be beautiful but preoccupied with image. Although she did not move to the UK deliberately for its culture, her insider–outsider perspective allowed her to appreciate cultural differences that were working out well for her:

How it changed me? More relaxed. Yes, stressed out more, working more maybe, relaxed in the way that I don't care you know, like I'm, I'm not, ages 13 years ago, would never walk out through the door with no makeup, because everybody would say you know like oh, she's sick, something wrong with her. Now I can you know, and even my mum, she moved as well with me to live in here, she would never work. Like works with us in the garden, oh we've run out of milk, pop in the shop. So she's with her gardening clothes can run to the shop, pick up the milk and come back. In Lithuania, you need to take a shower to to, change your clothes and go to the shop because the neighbour is going to see it. So in that case we are more relaxed, much more relaxed, which is good. Like I don't care you know just don't look at me if you don't like it. You know just I'm happy.

In this example, Julija makes an intergroup comparison to explain how the preoccupation with appearance she ascribed to her old home in Lithuania seems to her to be absent in her new life, which she describes as more relaxed, carefree, good and happy, despite working longer hours. Others, too, used this insider–outsider perspective to reflect on their engagement with different cultures. Chrysa valued the free national health service in the UK, and appreciated the discipline with which people queued up outside the bank. But she was also critical of both Greek and British culture: The former seemed to her to be living in the past, the latter poorly defined and not sufficiently cared for. She seems clear on what the defining characteristics of Greek culture are, acknowledging the less favourable ones too:

I feel that our history has destroyed Greece now because we proud ourselves of what we've given to the world and oh my god, we've given them all these things and you should be thankful of our democracy and philosophy and medicine blah blah blah. I feel like you know then nothing is happening, Greeks have become lazy you know, we've done our bit. So they can be very arrogant in that sense, we are the best, we know best.

Regardless of whether these qualities are attractive, Chrysa emphasizes the importance of a country having its defining characteristics, as well as the people understanding what is different and unifying within their culture. She sees this as lacking in the UK:

Chrysa If you were to ask me where is the culture in England, I'd be like actually, I think I know but I'm not quite sure, I'm not sure what British people associate themselves with. You know I feel like that there's not that sense of culture. I can tell you about Asian or Italy or Spain, I'm not quite sure about England, so that's one of the things that but that's different to what you asked but…

Interviewer But interesting.

Chrysa Yeah, I I just don't know what what is it. What is it that you feel is British culture? And and you ask and they will say you know, I'm I'm disappointed when people say we make tea and we stand in a queue I'm like no, no, no, that's not your culture, that's not what what is it, what is it, what is it you value, what you believe in.

Their status as insider–outsiders thus seems to have given participants a greater degree of cultural and intercultural awareness, from which they have a perspective on both (or all) of their ‘home’ cultures that eludes those who have never migrated. This awareness expressed itself in a differentiated view of national cultures rather than a straightforward and absolute preference for one or the other. Participants discussed old cultural preferences that persisted after migration, but also things they greatly admired in the culture of their new home. Markus, for example, describes how his cultural preferences make him permanently different from native British people:

But, if you say, what type of bread do you like or what type of butter do you like or what type of meat do you like, you still prefer the home meat. You cannot change that preference. And that has all something to do with ‘home’ I think. Because everything that is back home is much better than here. So I have to be very careful sometimes to not be too negative here because I find that you know, the bread is better and cars are better, blah, blah, blah.

For Markus, these preferences are embedded in a sense that he can never become British although he has lived in Britain for a very long time, feels happy with that arrangement, and appreciates his British friends. To Markus, this is quite natural (‘You cannot change that preference’). His status was, is, and always will be that of a guest. Nevertheless, his adopted role as a good guest seems to involve keeping his German culture quiet (‘I try to blend in rather than walk around in Lederhosen [laughs], you know?’), not challenging views with which he personally disagrees, and even defending British politics and British character when talking to others, which he states justifies his choice to be in the UK:

Markus … as soon as I am in Germany I defend very strongly what is happening here, you know, Thatcher, the football and driving on the left is better than driving on the right and stuff like that, you know?

Interviewer Uh huh.

Markus Whatever is being asked in Germany by friends and relatives about the UK, I, I find myself very strongly defending it, in a sense justifying my being here.

Kristina, meanwhile, explained how she never fully settled psychologically in the UK, but also admired a sense of patriotism and tradition that she never experienced in her own upbringing. Although she often uses superficially disparaging language when talking about these things (e.g. referring to the ‘stupid concept’ of the monarchy), she repeatedly talks about them as being good for the ‘health’ of a nation, even its ‘backbone’, and mentions in several places the metaphor of a flag to rally around in times of need or crisis. The absence of such a flag is something negative in her mind because it means that something important is missing.

The downside of it is when you don't have your flag and you feel like you're home everywhere. When you feel low in life it's so much harder. It's erm you don't have anything to weighing you down but you also don't have the support. It's amazing what I think English have and they don't realise having Queen as this imaginary thing which many just think what a ridiculous concept and no one thinks about it waking up in the morning. But when there are shaky moments or you want to celebrate or you mourn and she's there or you know you you have one of those celebrations, it's suddenly, there is you all sort of you know feel something it it connects nation without anyone thinking about it. It's ABSOLUTELY fantastic advantage. It's like backbone of of you know nation and I think most nations are so fascinated because they don't have it.

The flip side of this admiration is that Kristina feels that the Englishness she admires is being threatened by globalization. The failing of ‘global clever people’, in Kristina's view, is that they have cultivated a fascination with outside cultures and neglected the more local Englishness that she admires. Kristina makes clear that the idea of a disappearing Englishness comes from her husband, but her insider–outsider perspective on cultural change has made her adopt it:

I think that that sometimes English are surprised when immigrants sort of are those who … you find Brexit immigrants and it sort of doesn't make sense but they can see that what they loved most about England and they value because it doesn't exist anywhere else not especially the place they come from, that it's actually disappearing. My husband says there is no more England that that Englishness is disappearing … And in many ways it's a good thing, in many ways it's it's just horrible.

Notwithstanding the advantages of a migrant's perspective, being a ‘guest’ (and, by implication, not the host, landlord or owner) remains the participants' starting point and default position in the host society. They had chosen to move for the personal reasons discussed above, and there was no sense that the UK belonged to them. When Markus says that ‘the land is not my land. It is not my homeland’, he spells out what was implicit in many other accounts: Participants did not see themselves as ever being able to be ‘at home’ in the same way as British people without a migration background. For Chrysa, this leads to uncertainty about whether to stay in the UK or return to Crete. She expresses disappointment that her new home cannot live up to the ideal she held, while also having positive experiences which keep her in her new country:

So before it was always like oh nor sure do I stay, should have stayed at home, constantly. When I will go back to Greece, I really liked it and oh, I really want to stay here, that's it, I'm going to move. There have been at least I would say five times in the past 11 years that I was getting ready, I was like I'm going back, I'm going back. And you know starting rit‐, I was literally getting into it, I was in the process of contacting people, you know different uni's in Greece, what can I do, you know what where else can I work, what you know I was really getting into it. And then something will happen like good good usually something will happen as in yeah and then I was like, no no, I want to stay.

These feelings of being torn are unique to the migrant experience: For the move to be questioned and regretted, it must have happened in the first place. Kristina similarly described never (psychologically) moving to the UK and never (psychologically) ceasing to live in the Czech Republic:

Moving and sort of resolving those two places. And my husband in some argument erm told me which really took me aback, you NEVER moved here actually. And he's right in many ways, I'm refusing to to leave it when I realised that actually when I concentrate on my English and it improves, and I stop writing in Czech, I'm losing Czech, I panicked. And even if I make SO much less money than I would take different job here, erm … I'm just can't face I I feel like I can't afford to to let it go, that I know people who and it again, it's really depends how you know. I know people whose Czech is just absolute atrocious and I can't you know it it would really bother me to lose that language. So he's right, I I actually never stopped living there so I I'm making it for myself more complicated (laughs) than it is.

This is not as simple as feeling unhappy in the new place. There certainly were differences in how satisfied the participants felt with their current life arrangements; but even for those who liked where they were and had no urgent intention to leave, the sense of being different and having a home elsewhere was present. Lucie was settled in the UK, but it was also important to her to live near an airport so that travel between her old and new homes would be easy. She was grateful for the freedom of her British life and the kindness with which England had treated her, but also aware of being a guest:

I just always, ALWAYS have I have always felt that there was erm it didn't matter that I was from a different cul‐ country, I felt always very welcome and I've never had any negative experience of anybody making a comment or asking strange questions or being racist or in any way. I have been very lucky. Erm so I just always felt it didn't really matter that I was a foreigner and that that really helped. I think if you're in an environment when people let you feel it it's much harder.

In Lucie's case, then, the host country has not been a disappointment and she does not feel singled out as different. Still, in her own mind, her guest status is clearly reflected in feeling ‘welcome’, referring to herself as a ‘foreigner’, and acknowledging herself as a potential—even if not actual—target of racism. A sense of difference persists.

### Concern with feeling safe and welcome

The fourth cross‐case theme revolves around participants' concerns with feeling safe and welcome. Where this was not the case, efforts to make a new home were understood as more difficult or even futile.

Feelings of connection with family, friends and good neighbours were important in feeling safe and welcome. Alessia lived with her sister in London for a while, Chrysa lived with her sister and then a Cypriot friend. The majority of participants followed and lived with their spouses or romantic partners and/or met new partners after migration. The relationships did not always last, but were mentioned as a part of settling in and/or feeling at home. Lucie connected this explicitly to the feeling of safety that made her feel at home:

I suppose I grew older and more mature and I had [husband] who makes me feel very safe here so I think because I didn't feel I'm being threatened I've managed to calm down and properly look around and explore and enjoy England.

But new friends and neighbours in the new environment were also important. Examples already mentioned include Julija's local community and Lucie's experience of kindness from those she had met in the UK. Markus talked about friends whom he meets for golf, and Tomas about banter with work colleagues. Kristina made a lot of the support she felt from her allotment neighbours, even though they disagreed over Brexit:

So after Brexit, because we had proudly our ‘stay in Europe’ so everyone knew what we are and my neighbour I knew who wanted to leave. She came and again “I I know you'll love this hydrangea I I have a cutting for you.” She wanted to show me it doesn't matter, I LOST (laughs) that she wants you know. And this is erm this sort of compassion I found in allotment though they erm some people call me ‘that Polish woman,’ but when I'm in trouble they still come and help me (laughs).

Many participants felt that they belonged in diverse and multicultural environments, especially London. Participants who lived there often pointed to London's diversity and community as somehow different from the rest of the UK. David put this very clearly:

Here in London, why I said I feel like kind of home, is because London is so multi‐national, or multi‐ethnicity that you feel like there are so many ‘Frenchies’ in London, or so many Italian, or Spanish or Indians or whatever, so I guess this melting pot makes London feel like home for everybody.

Alessia also points towards a culture of openness and tolerance that made her feel at home when she arrived in the UK:

I don't know if I'm like idealising it a bit because it was eleven years ago, but I think it was, it was different. I think a lot has changed since then. But yeah, welcome in a sense of I never ever felt like unwanted or this place is not good for me because I'm different, never had any, never ever felt this way.

This, Alessia felt, changed with the Brexit referendum. The UK's departure from the EU, to her, suddenly imposed an unwanted outsider status on Europeans living in the UK. She suggests an undeserved loss of belonging, but that people like herself could regain control by using their previous migration experience to go and live elsewhere (implying that others do not have that luxury):

Imagine you lived in a country and you've done everything possible to be like a good citizen. And then all of a sudden you see that things are changing around you and you feel a little bit uncertain because you don't know what's going to happen with your job. You don't know what's going to happen if you don't get this citizenship and um … You might, it might happen that you don't have the same job opportunities as you had before and be treated as a citizen, a citizen of class B. Why? So why if I am valuable, I can find a job somewhere else, because we are, because we've been already, because we live already in another country, we're very flexible and we can easily, easily move somewhere else.

Similarly, Lucie talked about feeling welcome and respected ever since her arrival, and experiencing the referendum result as something that ‘really really shook the ground and now we just have to see if it settles’. She had not known British people with prejudices against immigrants, but the vote showed a wider political situation where she explicitly no longer felt safe and welcome:

I think just to feel, I think it was the immigration narrative and that and I I do appreciate that a lot of people, that wasn't the reason for why they voted leave, but they knew that that by by voting leave they would contribute to this narrative indirectly even though maybe it wasn't quite what they value, so I do think there was some kind of inner racism going on (laughs). Erm and just feeling not wanted really. Hmmm yeah, feeling unsafe.

These feelings of not being wanted or safe are the opposite of how she had felt previously within the UK, which indicates the enormity of how Brexit had impacted her. For Lucie, Brexit made her feel like an outsider when she hadn't been before, and which feels particularly difficult when she has worked hard and invested in the country.

Participants had different perspectives on this. Brexit was commonly characterized as exclusionary and incompatible with (participants' perceptions of) British values, but not always as a threat to participants' own sense of home. David did not feel personally impacted by Brexit because his identification as a Londoner allowed him to align himself with the Mayor of London's defence of multiculturalism and distance himself from its rejection. In London, he could still feel safe and welcome:

If anything I feel more Londoner and especially when I hear like Khan or like the Mayor of London, his speech after Brexit was brilliant erm regardless of your political view whatever, it was a good speech. And erm and I feel more more like a Londoner.

Markus referred to Brexit as ‘hurtful’ and ‘silly’, but not as a personal threat. Moreover, his role as a guest did not entitle him to comment on British decisions. Hanna feels secure enough because of her immediate environment in London and her settled status (‘there's no way I'm going to be forced to leave because I you know I got permission to stay here on my own, I wasn't married then’). For Francesca, the length of time she has been in the UK and her secure and stable home in London appear to be protective factors in her continuing to feel that London is home:

I think because I've been here quite a while and I am settled, I'm not still looking for the flat, for what career I should take and you know, my address on my passport is mine. I don't know I just and I registered yeah with the AIRE, which is like the Italian association for people residing abroad to like all this, I don't know, I just feel I've been here for so long that it didn't make me feel like oh, what's next, this is home, it has been home since 99, oh well, maybe not from the beginning but 2000, 2001. Yeah, it didn't really shift.

Tomas had experienced no detriment in his personal milieu and was happy to regard his colleagues' comments about Brexit as harmless jokes and banter. Although he had some concerns over his personal right to remain in the UK and talks about feeling very nervous about this for a while, he seems to have faith in Brexit itself bringing more prosperity, better opportunities, and higher regard for hard‐working immigrants. Having survived a personal odyssey for work, in which he persisted through numerous setbacks, he makes a distinction between immigrants such as himself, who come to the UK to work, and those whom he sees as benefit claimants who give all immigrants a bad name. His hope seems to be that Brexit will cause the latter to stay away and the former to be perceived in a better light:

I hope so there'll be a change after Brexit as well so that people are not coming here claiming the benefits and all them immigrants that are coming here and claiming it, is putting the wrong light on the other ones who come here and try to work it out, way up and just literally pay everything we can, bills, taxes and stuff like that, and still not being in the one big bag, naming it immigrant.

However, the momentousness of Brexit was generally acknowledged, and other participants described its impression on themselves as seismic. It was noted above how the event caused people to feel less safe and less wanted, and how this threatened the sense of home. Julija put this in particularly vivid terms:

In the morning, I woke up in the morning, I couldn't sleep all night, that day I felt like I'm not at home any more. I thought really it's not my home any more. Like really, and other people. Like most other people I said how do you feel as well? They said really, I don't belong here any more. You know just like, it's just really strange. Even the sun was a different colour. You know when just everything feels like so not mine any more. Like walking a hundred times on the streets, it's not mine any more. It was a really sad feeling.

In the immediate aftermath of the EU referendum, Julija and her family experienced xenophobic comments. For the first and only time in the interview, she uses the word ‘abuse’ (twice) to refer to the verbal treatment received by her and her mother. There is a vulnerability and insecurity being expressed here that is absent from Julija's usual confident way of dealing with situations. In her case, with the sense of feeling safe and welcome lost, an emotional home had been (temporarily) lost too. Notably, Julija's recovery from this was connected to feeling supported again:

It's, in some point it was like all these emotions and all this you know, people support and lots of people around me like you know who are coming here, they were very supportive as well. We even closed our [workplace] for a march, the anti‐Brexit march, because people said like oh, we're going to support that so it was just like we're supporting silently, all this. So it's just all this and like actions makes us feel better you know, if something goes on. And like yes, I started to like and the numbers yes almost 50% voted that they wanted to stay in the EU. You're starting to a bit like calm down.

The concern with being safe and welcome was thus a theme for all participants in that this was vital for their sense of home. But participants differed in how Brexit impacted this. For some, Brexit threatened or removed the sense of being safe and welcome; for others, the impact was mild or absent. This variability appeared to be connected to how participants reasoned about their sense of home. Feeling connected to the people close by, in an environment where they felt they belonged, was a key aspect of a sense of security.

## DISCUSSION

Starting from the idea that home is not just a place (Nowicka, 2007) and that the psychological, subjective sense of home among migrants involves both routes and roots (Ralph & Staeheli, 2011), we explored how this sense of home was experienced and understood by migrants. The British decision to leave the EU created a situation where immigrants' sense of home was especially important and in danger of being unsettled (Bueltmann & Bulat, 2021). Our participants talked about home, migration, and life as an EU citizen in the UK post‐Brexit, and we used IPA (Smith et al., 2022) to develop an analysis that would highlight any convergence in participants' accounts as well as honouring the uniqueness of each person's experience.

Our cross‐case experiential themes were about having more than one home, making and finding a new home after migration, being permanently different from the non‐migrant population, and having a concern with feeling safe and welcome. The notion of identity ran through all themes and interviews, supporting the idea that migration, acculturation, and the making of a new home involve building and rebuilding—or perhaps re‐imagining—one's identity (Romoli et al., 2022). Sometimes the new home was part of growing up, sometimes an important step in life or work. For our participants, their sense of home was bound up with who they were, including personal, national and place identities (see Proshansky, 1978; Tajfel & Turner, 1979).

Having more than one home was a central idea for all participants. It was understood variously as a privilege, a normal way of life or a source of uncertainty and unease; but always as a defining characteristic of being a migrant, which makes a sense of home necessarily complex and often complicated. The country of origin remained ‘home’ for all, but for nine of the ten participants the UK (or a specific place within it) was also ‘home’. Rather than being culturally homeless (see Hoersting & Jenkins, 2011), they navigated several homes psychologically and felt attached to several places simultaneously (see Gustafson, 2014). Elaborating on previous work that has connected acculturation to social identity (Brown & Zagefka, 2011), we can say that acculturation also seemed to involve the development of more complex place identities (Proshansky, 1978).

Often, different homes had different meanings. The current home was typically defined by practical points such as work opportunities and a family, and evaluated as a pleasant place to be if the physical and social surroundings were comfortable. The old home was sometimes cherished with a sense of nostalgia (Romoli et al., 2022; Smeekes & Jetten, 2019); on other occasions, participants acknowledged that the old home no longer existed in practical terms or had become the wrong place to be. Nevertheless, it remained present in these migrants' sense‐making of home.

Time was evidently important in supporting the transition (see Drinkwater & Garapich, 2015). However, there was also a more deliberate orientation towards particular ways of acculturating (see Berry, 1997). In their role of insider–outsiders, participants made comparisons and judgements about the old home(s), the new home, satisfaction with current arrangements and future plans. Personal qualities as a homemaker were important (see Romoli et al., 2022) and included, among other things, the ability to make cultural choices (see Barker, 2015). But the ability to relax and enjoy the new home also mattered. Holding on to traditions from the country of origin may enable a sense of familiarity within a new environment (Rabikowska, 2010). But participants also incorporated new traditions, objects, tastes, values and aspects of themselves, suggesting choice in the acculturation process (Barker, 2015). The ‘planting of new seeds’ was accompanied by a ‘keeping of old roots’, in line with the familiar idea that a positive orientation towards both the culture of origin and the host culture is beneficial (Berry et al., 2006). This balancing and hybridisation of the old and new homes was still in progress and may never reach resolution (see George & Fitzgerald, 2012). It seems to be part of the challenges involved in having more than one home.

The conscious and effortful process of making the new home (see Shamma et al., 2022) was sometimes accompanied by more serendipitous events such as having kind neighbours or work colleagues. We found homemaking facilitated by practical factors such as the presence of loved ones, jobs, a grounding place (such as a house or a pleasant environment), but also a sense of comfort or admiration towards the host culture. This helped create the moves in the first place and then made them more permanent. For many, this involved developing the lives they hoped for, and therefore imagining a future in the new home. If contact and participation are essential ingredients of integration (see Berry, 1997), the process of temporary moves becoming permanent homes seemed to involve things that facilitated this contact and participation.

Nevertheless, the sense of being a guest—a privileged position compared with outsiders or newcomers, though submissive towards those who are more established (see McKinlay & McVittie, 2007)—never went away for some. We did not find a sense of psychological ownership of the UK (Brylka et al., 2015; Verkuyten & Martinovic, 2017). Being permanently different from the natives was these participants' starting point and default position, which is arguably an essentialist view (see Rothbart & Taylor, 1992), but perhaps also a corollary of holding on to more than one home and integrating, rather than assimilating, into a new culture (Berry, 1997; Bourhis et al., 1997).

This research illustrates the importance of the receiving context (Berry, 1997; Schwartz et al., 2010) in reconstructing home (see Miller, 2019). A sense of home was connected to a sense of feeling safe and welcome (see Romoli et al., 2022). Friendly neighbours and communities, as well as diversity (see Lulle et al., 2018), helped. In contrast, Brexit upset the sense of home (Guma & Jones, 2019; Hall et al., 2022; Lulle et al., 2018; McGhee et al., 2017). Some considered the referendum to have had an impact on feeling wanted and on their thinking about their future in the UK (see Tyrrell et al., 2019). Others seemed more secure, feeling that the referendum had not taken away their own sense of home. This suggests that the perceived orientation of the host society is indeed a strong influence (see Berry, 1997), but that subjective lived experiences may differ and have different consequences for acculturation attitudes (see Celeste et al., 2014) and the sense of home.

### Reflection and limitations

IPA enabled us to investigate home and place attachment as feeling and meaning (rather than measurement or argument). Although IPA's impact has been greatest in British health psychology where it originates (Smith, 2011), our study illustrates its usefulness in social psychology, too. The experience of migration, the subjective sense of home, living through Brexit as an EU citizen in the UK and the identity changes associated with these are all eminently suitable topics for IPA with its focus on subjective lived experience. IPA's idiographic commitment allowed us to understand each participant's unique situation and understanding, but also the important commonalities between people that point towards essential qualities of the phenomenon (Smith et al., 2022). The present paper thus contributes to establishing IPA as a method to examine ‘lived identity’ in social psychology (see Hunt et al., 2021; Warren & Nigbur, 2024): Where people reason about how their social identities affect, and are affected by, interactions with the cultural environment—including the settings that they regard as home—IPA is the ideal method to investigate this sense‐making (see also de Visser & Smith, 2007; Huff et al., 2019).

Certainly, our participants are neither representative of all EU citizens in the UK nor of their countries of origin. They all had different previous homes and idiosyncratic histories. But they were homogeneous not just in being EU citizens resident in the UK during Brexit, but also in some psychological challenges. The memory of the past, the experience of migration and the reconstruction of identity and relationships were confirmed as major elements in migrants' understandings of their psychological home (see Romoli et al., 2022). While generalizability is thus neither possible nor intended by our methodological approach, these insights may be transferable (see Smith et al., 2022) to other EU immigrants to the UK, or even to other migration contexts altogether. Future research could focus on the different context of Scotland, where a majority of voters rejected Brexit (Sigona & Godin, 2019), and on how migrants avoid cultural homelessness (Hoersting & Jenkins, 2011).

## CONCLUSION

Migration researchers have called for more qualitative work to illuminate processes such as acculturation, adaptation and a sense of home through the analysis of first‐hand accounts, with sensitivity to local and personal contexts (Chirkov, 2009; Romoli et al., 2022). Our study has addressed this by using IPA (Smith et al., 2022) to examine the lived experiences of EU citizens in England during Brexit. We believe that our study demonstrates the usefulness of this approach in studying how migration and acculturation (Berry, 1997), social identity (Tajfel & Turner, 1979) and place identity (Proshansky, 1978) are lived, experienced and understood. Without neglecting the psychologically important individuality of each migrant—and indeed building on precisely this idiographic perspective—we have identified some convergence that may help understand migration, acculturation and identity more generally. If making a new emotional home is an active and effortful process, and neither the old home nor the difference from the native population ever goes away psychologically, this strengthens the case for integration (Berry, 1997) as a promising acculturation strategy in a migration context. Although the migrants in our study negotiated psychological tensions in their sense of home, this was not a zero‐sum game that could be resolved by assimilation or a return to the old home. Regardless of their commitment to the new home, their situation and lived experience remained special.

## AUTHOR CONTRIBUTIONS

**Kate Foxwell:** Conceptualization; data curation; formal analysis; writing – original draft; methodology; investigation; project administration; writing – review and editing. **Sarah Strohmaier:** Conceptualization; data curation; formal analysis; writing – original draft; methodology; investigation; writing – review and editing; project administration. **Fergal Jones:** Conceptualization; writing – original draft; supervision; writing – review and editing. **Dennis Nigbur:** Data curation; formal analysis; writing – original draft; methodology; writing – review and editing; project administration; investigation.

## CONFLICT OF INTEREST STATEMENT

The authors have no conflict of interest to declare.

## Data Availability

The data that support the findings of this study are available on request from the corresponding author. The data are not publicly available due to privacy or ethical restrictions.
